# Potassium-increasing regimens in patients at high risk of ventricular arrhythmias: a prespecified observational analysis of the randomized POTCAST trial

**DOI:** 10.1093/europace/euag272

**Published:** 2026-10-06

**Authors:** Elisabeth Margrethe Danielsen, Emil Anton Frandsen, Ulrik Christian Gudbrand Winsløw, Chaoqun Zheng, Tharsika Sakthivel, Kasper Karmark Iversen, Helle Skovmand Bosselmann, Niels Risum, Henning Bundgaard, Christian Jøns

**Affiliations:** Department of Cardiology, The Heart Centre, Copenhagen University Hospital—Rigshospitalet, Blegdamsvej 9, Copenhagen Ø 2100, Denmark; Department of Cardiology, The Heart Centre, Copenhagen University Hospital—Rigshospitalet, Blegdamsvej 9, Copenhagen Ø 2100, Denmark; Department of Cardiology, The Heart Centre, Copenhagen University Hospital—Rigshospitalet, Blegdamsvej 9, Copenhagen Ø 2100, Denmark; Department of Cardiology, The Heart Centre, Copenhagen University Hospital—Rigshospitalet, Blegdamsvej 9, Copenhagen Ø 2100, Denmark; Department of Cardiology, The Heart Centre, Copenhagen University Hospital—Rigshospitalet, Blegdamsvej 9, Copenhagen Ø 2100, Denmark; Department of Cardiology, Copenhagen University Hospital—Herlev and Gentofte, Herlev Ringvej 75, Herlev 2730, Denmark; Department of Clinical Medicine, University of Copenhagen, Blegdamsvej 3B, Copenhagen N 2200, Denmark; Department of Cardiology, Zealand University Hospital—Roskilde, Sygehusvej 10, Roskilde 4000, Denmark; Department of Cardiology, The Heart Centre, Copenhagen University Hospital—Rigshospitalet, Blegdamsvej 9, Copenhagen Ø 2100, Denmark; Department of Cardiology, The Heart Centre, Copenhagen University Hospital—Rigshospitalet, Blegdamsvej 9, Copenhagen Ø 2100, Denmark; Department of Clinical Medicine, University of Copenhagen, Blegdamsvej 3B, Copenhagen N 2200, Denmark; Department of Cardiology, The Heart Centre, Copenhagen University Hospital—Rigshospitalet, Blegdamsvej 9, Copenhagen Ø 2100, Denmark; Department of Clinical Medicine, University of Copenhagen, Blegdamsvej 3B, Copenhagen N 2200, Denmark

**Keywords:** Hypokalemia, Hyperkalemia, Implantable cardioverter-defibrillator, Mineralocorticoid receptor antagonists, Potassium supplements, Sudden cardiac death

## Abstract

**Aims:**

The POTCAST trial (*n* = 1200) showed that targeting high-normal plasma potassium levels reduced ventricular arrhythmias in patients with implantable cardioverter-defibrillators (ICDs). We compared changes in plasma potassium and described outcomes across the three regimens used to increase plasma potassium.

**Methods:**

We studied 600 control participants and 572 intervention participants who completed potassium up-titration in POTCAST. Participants in the intervention arm were categorized according to treatment after plasma-potassium up-titration into (i) mineralocorticoid-receptor antagonists (MRAs) monotherapy, (ii) potassium-supplement monotherapy, or (iii) combination therapy with both. The primary endpoint was a composite of sustained ventricular arrhythmia, appropriate ICD therapy, hospitalization for arrhythmia or heart failure, or all-cause mortality. Safety outcomes included death and electrolyte/renal disturbances.

**Results:**

After 6 months, plasma potassium increased from 4.12 to 4.27 mmol/L (*P* = 0.004) with MRA, from 3.99 to 4.24 mmol/L (*P* < 0.001) with potassium supplements, and from 3.98 to 4.34 mmol/L (*P* < 0.001) with combination therapy. The primary endpoint occurred in 29% (*n* = 175) of controls, 22% (*n* = 16) in the MRA group, 21% (*n* = 31) in the potassium supplements group, and 22% (*n* = 72) in the combination therapy group (*P* = 0.8 between regimens). Hypokalemia occurred in 13% of controls and in 3, 9, and 5% of participants receiving MRA, potassium supplements, and combination therapy, respectively. Hyperkalemia occurred in 0.2% of controls and in 0, 3, and 4% of participants receiving MRA, potassium supplements, and combination therapy (*P* > 0.1 between regimens).

**Conclusion:**

Treatment with MRA, potassium supplements, or both increased potassium levels. All regimens were associated with numerically lower endpoint rates than controls. We detected no statistically significant differences in endpoints or safety between regimens.

## Structured Graphical abstract

**Structured Graphical abstract:**
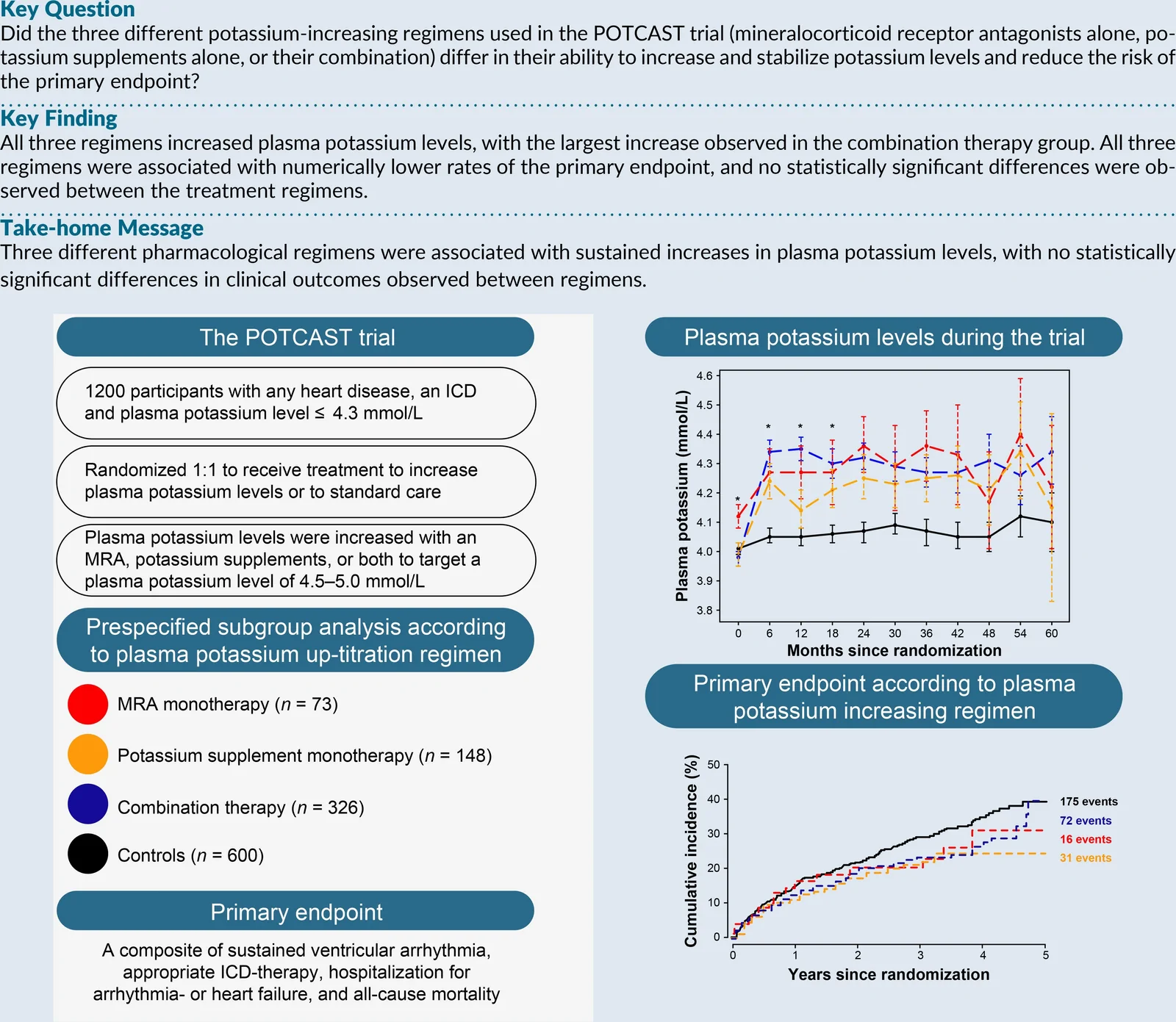
Graphical overview of the POTCAST subgroup analysis. The present prespecified POTCAST substudy included all 600 control participants and 572 intervention participants who completed plasma potassium up-titration (28 intervention participants did not start up-titration e.g. due to withdrawal). The three active potassium-increasing regimens were compared with each other and with the control group: MRA monotherapy (*n* = 73), potassium supplement monotherapy (*n* = 148), combination therapy (*n* = 326), and controls (*n* = 600). After attempted up-titration, 25 participants were not receiving active study medication, e.g. due to intolerable side effects. Findings from these participants are not reported in this subgroup analysis. * = *P* < 0.05 between intervention regimens. MRA denotes mineralocorticoid receptor antagonist; ICD denotes implantable cardioverter-defibrillator.

## Introduction

Potassium is essential for maintaining normal cardiac function, and disturbances in plasma potassium levels are strongly associated with adverse outcomes in patients with cardiovascular disease.^1–3^ Both hypokalemia and hyperkalemia increase mortality risk, and even levels within the low-normal range (3.5 to 4.0 mmol/L) are associated with excess risk of ventricular arrhythmias and sudden cardiac death.^4–7^

Observational studies have indicated that plasma potassium levels in the high-normal range (4.5 to 5.0 mmol/L) reduce mortality.^8^ These observational findings were recently investigated prospectively in the randomized clinical POTCAST trial *The Targeted Potassium Levels to Decrease Arrhythmia Burden in High-Risk Patients With Cardiovascular Disease*. The study demonstrated that targeting plasma potassium levels to the range between 4.5 and 5.0 mmol/L through potassium-rich dietary counseling and mineralocorticoid receptor antagonists (MRAs), potassium supplements or the combination of the two, significantly reduced the incidence of the composite outcome consisting of sustained ventricular tachycardia (VT), appropriate implantable cardioverter-defibrillator (ICD) therapy, unplanned hospitalization >24 h for arrhythmia or heart failure leading to a change in drug or invasive treatment, and all-cause mortality without increasing the risk of hyperkalemia, hypokalemia, or renal failure.^9^

Several randomized landmark trials have demonstrated that MRA treatment improves survival in patients with heart failure.^10–12^ This effect has been related to the inhibition of maladaptive neurohormonal pathways,^10–12^ but MRAs also increase plasma potassium levels.^12^ Although the MRA-induced rise in plasma potassium level has traditionally been considered a safety concern, the novel findings from the POTCAST trial suggest that the elevated plasma potassium levels *per se* may contribute to the observed clinical benefits. If this is the case, an increase in plasma potassium levels induced by other means but MRA treatment, e.g. potassium supplements, would also be considered beneficial. We therefore conducted this prespecified observational analysis to characterize plasma potassium changes and examine clinical and safety outcomes across the three regimens used in the intervention group.

## Methods

### Study design and patient selection

The POTCAST trial was an open-label, randomized controlled trial conducted at three centers in Denmark where ICDs were implanted. The study design^13^ and main results^9^ have been published previously. In brief, the POTCAST trial randomized 1200 participants with any heart disease and treated with an ICD and a baseline plasma potassium level of no more than 4.3 mmol/L, and with a preserved renal function defined as a baseline estimated glomerular filtration rate (eGFR) of more than 30 mL/min/1.73 m^2^. The intervention was to increase plasma potassium levels in addition to standard care, while control participants received standard care only. Participants were enrolled from March 2019 through September 2024.

The intervention group received counselling on potassium-rich diet and treatment with MRAs, potassium supplements, or a combination of the two, according to a protocolized potassium-optimization strategy targeting plasma potassium levels between 4.5 and 5.0 mmol/L (see Supplementary material online, *Figure S1*). The baseline visit required in-person attendance for information, informed consent, and baseline examinations. The remaining up-titration phase and subsequent follow-up were conducted by local blood sampling followed by telephone consultations. MRA therapy was recommended as first-line therapy followed by potassium supplements if target levels were not achieved, unless MRAs were not tolerated or contraindicated, or if participants expressed a preference for potassium supplements. The maximum daily MRA dose was 100 mg for spironolactone, 50 mg for eplerenone, and 4500 mg for potassium supplements. Dose adjustments were guided by the obtained plasma potassium level and predefined safety parameters, including plasma creatinine levels and blood pressure, and down-titration was mandated if thresholds were exceeded, as described in Supplementary material online, *Figure S1*. Once participants reached target plasma potassium levels, maximum study doses, or were unable to further up-titrate due to adverse effects or patient refusal of further medication, follow-up continued every 6 months with laboratory testing, electrocardiogram, and ICD interrogation. Up-titration was completed in 572 participants in the POTCAST trial, and these were included in the present analysis. The study population for this prespecified analysis therefore comprised 1172 participants (600 control and 572 intervention).

### Exposure and outcomes

For this prespecified subgroup analysis, participants in the intervention group, who completed the up-titration phase, were classified into three plasma potassium-increasing regimens according to their study medication status at the end of the up-titration: MRA as monotherapy, potassium supplement as monotherapy, or combination therapy of both MRA and potassium supplements. Consequently, allocation to the individual treatment regimens was not randomized, and comparisons between regimens were observational. Findings from participants who did not receive study medication after completing up-titration are not reported.

The primary endpoint in the POTCAST trial was a composite of first occurrence of sustained VT (>125 bpm >30 s documented on an electrocardiogram or an electrogram), appropriate ICD therapy, unplanned hospitalization >24 h for arrhythmia or heart failure leading to a change in drug or invasive treatment, and all-cause mortality. The safety endpoint was a composite of the first occurrence of unplanned hospitalization >24 h due to hypokalemia, hyperkalemia, or renal failure, and all-cause mortality. Hypokalemia was defined as at least one plasma potassium measurement <3.5 mmol/L and hyperkalemia as at least one measurement ≥5.5 mmol/L during follow-up visits. All suspected study endpoints were adjudicated by an independent Endpoint Committee blinded to randomized treatment allocation, plasma potassium levels, and treatment regimen. In this substudy, we examined the occurrence of the primary endpoint and safety endpoints across the three treatment regimens and also compared them with controls.

### Statistical analyses

Continuous variables are reported as means ± standard deviations and are compared using the one-way analysis of variance (ANOVA). Categorical variables are reported as frequencies and percentages and compared using the χ^2^ test or Fisher’s exact test as appropriate.

Changes in plasma potassium levels from baseline to 6 months within treatment regimens were assessed using the paired *t*-tests, and subgroup proportions reaching high-normal plasma potassium levels were compared using the χ^2^ test. Changes in plasma potassium and creatinine levels over time were analyzed in a linear mixed model with time and treatment allocation and their interaction as fixed effects and with a random effect for each participant. The models included all available measurements. Missing values were not imputed.

The risks of the primary composite endpoint and safety endpoint were estimated using Cox proportional hazards models, reported as hazard ratios (HRs) with 95% confidence intervals (CI). For the individual components of the primary composite endpoint and the composite safety endpoint, the risk of all-cause mortality was analysed using Cox proportional hazards models, whereas the risks of the other components were assessed using cause-specific Cox proportional hazards models, treating all-cause mortality as a competing event. Multivariable Cox proportional hazards models were adjusted for baseline plasma potassium level, eGFR <60 mL/min/1.73 m^2^ at baseline, baseline use of potassium supplements, an MRA, or a sodium-glucose cotransporter 2 inhibitor, ICD indication, and previous VT. The assumption of proportionality was tested using the cumulative score process test. As a violation of this assumption was detected for previous VT, the Cox proportional hazards models were stratified for previous VT. In the primary analysis, the risk of the primary endpoint was estimated for each of the three plasma potassium-increasing regimens, defined at the completion of up-titration, compared with the control group and across the three intervention regimens, irrespective of any subsequent treatment crossovers. Participants in the control group entered the analysis on the day of randomization, whereas the time origin for participants in the intervention group was defined as the day of completed plasma potassium up-titration. Patients were considered at risk from time origin until the participant died, the participant withdrew, or until administrative censoring at the end of the trial (31 March 2025). To address the risk of immortal time bias in the primary analysis, two sensitivity analyses were performed. In the first, a landmark time of 85 days was chosen corresponding to the median duration of up-titration in the intervention group. In the second, all participants entered the study on the day of randomization with treatment exposure as a time-varying variable, thereby also accounting for cross-overs during the trial. Cumulative incidences of the primary composite endpoint and all-cause mortality were assessed using the Kaplan–Meier estimator. For the remaining components of the primary composite endpoint and the components of the safety endpoint, cumulative incidences were assessed using the Aalen–Johansen estimator. Missing data on clinical data, device data, and hospitalizations were very rare. A *P*-value < 0.05 was considered statistically significant. Analyses were performed using R statistical software, version 4.3.2

## Results

### Baseline characteristics of the participants

A total of 600 participants were assigned to the control group, and 572 participants in the intervention group completed plasma potassium up-titration (see Supplementary material online, *Figure S2*). The baseline characteristics of the control group and the three intervention regimens are presented in *Table 1*. In the intervention group, the mean age was 62.7 ± 11.9 years, and 20% of participants were female. Baseline characteristics were generally well-balanced between groups. However, participants in the MRA monotherapy regimen had significantly higher plasma potassium levels at baseline, compared to the other intervention regimens and controls (*P* < 0.001 for all pairwise comparisons). Baseline plasma potassium levels were similar in the two other regimens. Across all three intervention regimens, participants receiving either study medication at baseline were more likely to have undergone further up-titration with the same drug class.

**Table 1:** Baseline characteristics of the participants

	Controls (*n* = 600)	MRA monotherapy (*n* = 73)	Potassium supplement monotherapy (*n* = 148)	Combination therapy (*n* = 326)	*P*-value
Age, years	62.8 (11.8)	63.2 (11.2)	62.5 (13.5)	62.6 (11.2)	0.972
Female sex, *n* (%)	120 (20.0)	9 (12.3)	33 (22.3)	64 (19.6)	0.366
Left ventricular ejection fraction, %	43 (14)	42 (12)	46 (13)	43 (13)	0.088
New York Heart Association functional class, *n* (%)					
I	354 (59.0)	42 (57.5)	93 (62.8)	188 (57.7)	0.752
II	214 (35.7)	28 (38.4)	49 (33.1)	117 (35.9)	
III	32 (5.3)	3 (4.1)	6 (4.1)	21 (6.4)	
IV	0	0	0	0	
ICD for secondary prevention, *n* (%)	334 (55.7)	40 (54.8)	95 (64.2)	181 (55.5)	0.269
Previous ICD therapy, *n* (%)	160 (41.0)	26 (50.0)	45 (41.3)	97 (44.7)	0.568
Previous ventricular tachycardia, *n* (%)	390 (65.0)	52 (71.2)	109 (73.6)	217 (66.6)	0.198
**Medical history at baseline**					
*Ischemic heart disease and cardiac risk factors*					
Ischemic heart disease, *n* (%)	302 (50.3)	42 (57.5)	65 (43.9)	157 (48.2)	0.249
Acute myocardial infarction, *n* (%)	227 (37.8)	34 (46.6)	48 (32.4)	108 (33.1)	0.097
Heart failure, *n* (%)	385 (64.2)	50 (68.5)	85 (57.4)	227 (69.6)	0.061
Atrial fibrillation, *n* (%)	191 (31.8)	23 (31.5)	56 (37.8)	101 (31.0)	0.491
Hypertension, *n* (%)	320 (53.3)	38 (52.1)	81 (54.7)	160 (49.1)	0.581
Diabetes mellitus, *n* (%)	113 (18.8)	13 (17.8)	25 (16.9)	62 (19.0)	0.945
Renal failure, *n* (%)	59 (9.8)	2 (2.7)	24 (16.2)	36 (11.0)	0.017
*Nonischaemic cardiomyopathy and primary arrhythmia*					
Dilated cardiomyopathy, *n* (%)	90 (15.0)	10 (13.7)	26 (17.6)	67 (20.6)	0.156
Arrhythmogenic ventricular cardiomyopathy, *n* (%)	36 (6.0)	0 (0.0)	6 (4.1)	27 (8.3)	0.034
Hypertrophic cardiomyopathy, *n* (%)	34 (5.7)	3 (4.1)	10 (6.8)	15 (4.6)	0.738
Primary arrhythmia, *n* (%)	74 (12.3)	12 (16.4)	21 (14.2)	34 (10.4)	0.439
Non-ischaemic heart failure, unknown phenotype, *n* (%)	61 (10.2)	4 (5.5)	11 (7.4)	24 (7.4)	0.307
Other diagnoses, *n* (%)	21 (3.5)	2 (2.7)	10 (6.8)	9 (2.8)	0.173
**Medications at baseline**					
Potassium supplements, *n* (%)	163 (27.2)	4 (5.5)	42 (28.4)	84 (25.8)	0.001
Mineralocorticoid receptor antagonists, *n* (%)	229 (38.2)	40 (54.8)	4 (2.7)	178 (54.6)	<0.001
ACE inhibitor/ARB/ARNi, *n* (%)	427 (71.2)	54 (74.0)	97 (65.5)	248 (76.1)	0.107
Beta blocker, *n* (%)	485 (80.8)	58 (79.5)	110 (74.3)	262 (80.4)	0.359
SGLT-2 inhibitors, *n* (%)	99 (16.5)	11 (15.1)	11 (7.4)	73 (22.4)	0.001
Loop diuretics, *n* (%)	141 (23.5)	11 (15.1)	36 (24.3)	90 (27.6)	0.136
Thiazides, *n* (%)	51 (8.5)	5 (6.8)	12 (8.1)	28 (8.6)	0.966
**Laboratory findings at baseline**					
Plasma potassium, mmol/L	4.01 (0.24)	4.12 (0.17)	3.99 (0.24)	3.98 (0.24)	<0.001
Estimated glomerular filtration rate, mL/min/1.73 m^2^	77.5 (14.0)	78.4 (11.9)	73.8 (17.2)	76.9 (13.9)	0.031
Estimated glomerular filtration rate <60, *n* (%)	85 (14.2)	5 (6.8)	38 (25.7)	50 (15.3)	0.001

Baseline characteristics of the participants. Values are presented as mean ± standard deviation or number (%) of participants. ACE denotes angiotensin-converting enzyme, ARB denotes angiotensin II receptor blockers, ARNi denotes angiotensin receptor–neprilysin inhibitor, ICD denotes implantable cardioverter-defibrillator, MRA denotes mineralocorticoid receptor antagonist, and SGLT-2 denotes sodium-glucose cotransporter 2.

### Plasma potassium up-titration

At baseline, 272 participants (48%) were naive to both MRAs and potassium supplements; 169 participants (30%) were treated with an MRA; 78 participants (14%) were treated with potassium supplements; and 53 participants (9%) were treated with an MRA and potassium supplements.

At completion of up-titration, 73 (13%) received MRA as monotherapy, 148 (26%) received potassium supplements as monotherapy, and 326 (57%) participants received the combination therapy regimen (see Supplementary material online, *Figure S3* and *Table 2*).

**Table 2:** Up-titration according to baseline medication

	No. of visits to complete up-titration	Post up-titration regimen			
		MRA monotherapy (*n* = 73)	Potassium supplement monotherapy (*n* = 148)	Combination therapy (*n* = 326)	No treatment (*n* = 25)
Baseline treatment					
Naive to both study medications (*n* = 272)	4.5 ± 2.0	31 (11)	104 (38)	113 (42)	24 (9)
MRA (*n* = 169)	3.6 ± 1.9	38 (22)	2 (1)	129 (76)	0 (0)
Potassium supplements (*n* = 78)	4.4 ± 1.9	2 (3)	40 (51)	35 (45)	1 (1)
MRA and potassium supplements (*n* = 53)	3.8 ± 1.8	2 (4)	2 (4)	49 (92)	0 (0)

Visits are the number of visits required to complete up-titration and are reported as mean ± standard deviation. Other values are shown as number (%) of participants. MRA denotes mineralocorticoid receptor antagonist.

The most frequent reasons for not receiving potassium supplements in the MRA monotherapy regimen group were reaching target levels without requiring potassium supplements (45%) or due to adverse effects from potassium supplements (34%). In the potassium supplement monotherapy regimen, the most frequent reasons for not receiving an MRA were adverse effects (39%) and participant preference (36%). Supplementary  *Tables 1 and 2* summarize reasons for completing up-titration with only one of the study drugs, and Supplementary material online, *Table S3* presents adverse events leading to down-titration or discontinuation of study medication during up-titration.

The mean number of up-titration visits differed according to baseline medication: Participants naive to any of the study medications required more up-titration visits than those already receiving an MRA at baseline (4.5 visits in participants naive to study medication vs. 3.6 visits in participants receiving an MRA alone, *P* < 0.001; and vs. 3.8 visits in participants in combination therapy at baseline, *P* = 0.015) (*Table 2*). Similarly, participants only receiving potassium supplements at baseline required more up-titration visits than participants receiving an MRA at baseline (4.4 visits vs. 3.6 visits, *P* = 0.004). The most common reason for finalizing the up-titration phase was reaching the target plasma potassium level of 4.5 to 5.0 mmol/L, which was achieved in 249 (44%) of all participants. Reasons for completing up-titration are presented in *Table 3*, and dose adjustments are detailed in *Table 4*. Only 52 participants (9%) completed up-titration by reaching maximal allowed study dosages without reaching target plasma potassium levels.

**Table 3:** Reasons for completing up-titration according to baseline medication

	Reason for completing up-titration				
	Reaching target plasma potassium levels	Maximum study doses without reaching target	Adverse events	Patient refused further medication	Other
Baseline treatment					
Naive to both study medications (*n* = 272)	108 (40)	21 (8)	69 (25)	43 (16)	31 (11)
MRA (*n* = 169)	88 (52)	21 (12)	38 (22)	10 (6)	12 (7)
Potassium supplements (*n* = 78)	36 (46)	2 (3)	21 (27)	13 (17)	6 (8)
MRA and potassium supplements (*n* = 53)	17 (32)	8 (15)	18 (34)	5 (9)	5 (9)

Values are shown as number (%) of participants. Reasons for completion of up-titration are categorized as reaching target plasma potassium levels, maximum study doses reached without reaching target plasma potassium level, adverse events, patient refusal of further medication, and other reasons.

**Table 4:** Dose adjustments of MRAs and potassium supplements during the up-titration phase

		Post up-titration regimen		
		MRA monotherapy (*n* = 73)	Potassium supplement monotherapy (*n* = 148)	Combination therapy (*n* = 326)
Baseline treatment				
Naive to both study medications (*n* = 272)	MRA	+42.7 (±25.8)	–	+ 54.8 (±28.8)
	Potassium	–	+ 3626 (±1177)	+3378 (±1300)
MRA (*n* = 169)	MRA	+17.8 (±23.7)	−12.5 (±0)	+ 12.4 (±18.3)
	Potassium	–	+3750 (±1061)	+3256 (±1273)
Potassium supplements (*n* = 78)	MRA	+25.0 (±0)	–	+45.4 (±27.0)
	Potassium	−1698 (±781)	+2390 (±1102)	+1892 (±1546)
MRA and potassium supplements (*n* = 53)	MRA	+25.0 (0)	−25 (±0)	+14.8 (±26.4)
	Potassium	−1875 (±530)	+1125 (±530)	+2101 (±1113)

Values are expressed as mean change ± standard deviation in mg. Dose adjustments reflect the net change in prescribed dose from baseline to the end of the up-titration. Results are presented according to baseline treatment status and final regimen allocation after up-titration

### Plasma potassium changes during the study period

Plasma potassium increased significantly from baseline to 6 months follow-up in all intervention regimens: from 4.12 to 4.27 mmol/L; *P* = 0.004 in the MRA monotherapy group, from 3.99 to 4.24 mmol/L; *P* < 0.001 in the potassium monotherapy group, and from 3.98 to 4.34 mmol/L; *P* < 0.001 in the participants in the combination regimen. The increase in plasma potassium levels from baseline to s6 months was significantly higher in participants in the combination regimen compared to both participants in the potassium supplement monotherapy group (difference in increase: 0.11 mmol/L, 95% CI, 0.03 to 0.18, *P* = 0.005) and compared to participants in the MRA monotherapy group (difference in increase: 0.22 mmol/L, 95% CI, 0.12 to 0.32, *P* < 0.001). Adjusted for baseline plasma potassium levels, the increase with combination therapy remained significantly higher compared to the two monotherapy regimens with adjusted differences of 0.10 mmol/L (95% CI, 0.03 to 0.17, *P* = 0.004) compared to potassium monotherapy and 0.11 mmol/L (95% CI, 0.02 to 0.20, *P* = 0.014) compared to MRA monotherapy.

The change in plasma potassium levels from 6 to 12 months differed significantly between the potassium monotherapy group and combination therapy (*P* = 0.019) with a decrease in plasma potassium levels in the potassium supplement monotherapy group (*Figure 1* and Supplementary material online, *Table S4*). However, at month 18, plasma potassium levels had stabilized in the potassium monotherapy group, and there were no detectable differences in plasma potassium levels over time between the three intervention groups during the rest of the study (MRA monotherapy vs. potassium monotherapy, *P* = 0.181; MRA monotherapy vs. combination therapy, *P* = 0.334; potassium monotherapy vs. combination therapy, *P* = 0.502). The proportion of participants reaching the target plasma potassium level during the trial is illustrated in Supplementary material online, *Figure S4*.

**Figure 1:**
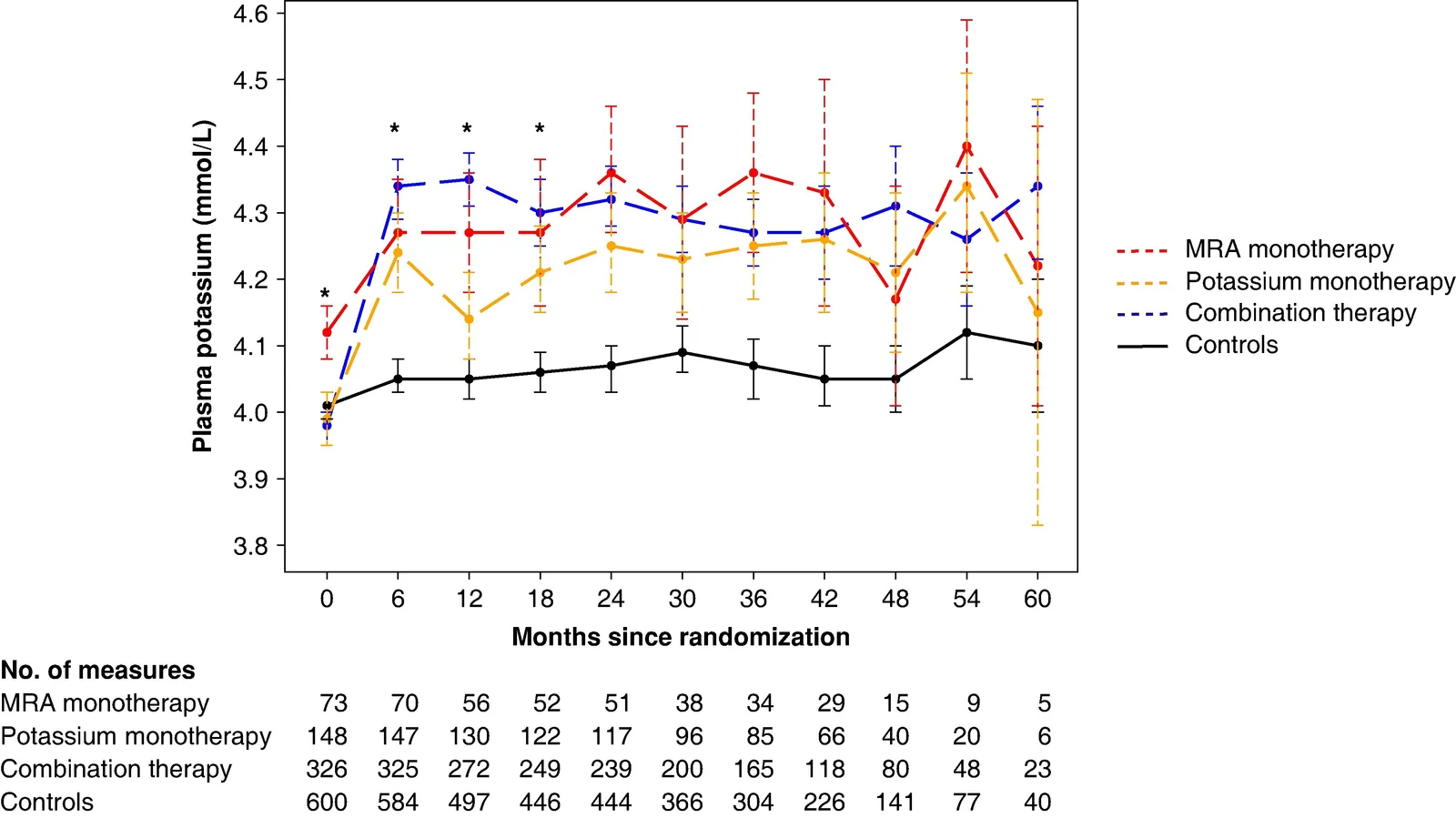
Potassium levels during the study by treatment regimen. Mean plasma potassium levels with 95% CIs during the study: MRA monotherapy (dashed red), potassium supplement monotherapy (dashed orange), combination therapy (dashed blue), and controls (solid black). * = *P* < 0.05 between intervention regimens. MRA denotes mineralocorticoid receptor antagonist.

### Endpoints

The median follow-up was 3.3 years (interquartile range (IQR): 2.2 to 4.1) in the control group compared to 3.2 years (IQR: 1.0 to 3.8) in the MRA monotherapy group, 3.3 years (IQR: 2.1 to 4.1) among participants in potassium supplement monotherapy, and 3.0 years (IQR: 1.9 to 3.9) in the combination therapy group. During this period, the primary composite endpoint occurred in 175 participants (29%) in the control group, in 16 participants (22%) in MRA monotherapy, in 31 participants (21%) with potassium supplement monotherapy, and in 72 participants (22%) in the combination therapy group (*Figure 2A*), with no detectable difference between intervention regimens (*P* = 0.8). The distribution of the individual components of the composite outcome across regimens is shown in *Figure 2B–E*. In total, 96% of the endpoints shown in *Figure 2C* were appropriate ICD therapies. The individual components show broadly consistent patterns, but the analysis was not powered to compare individual components between regimens.

**Figure 2:**
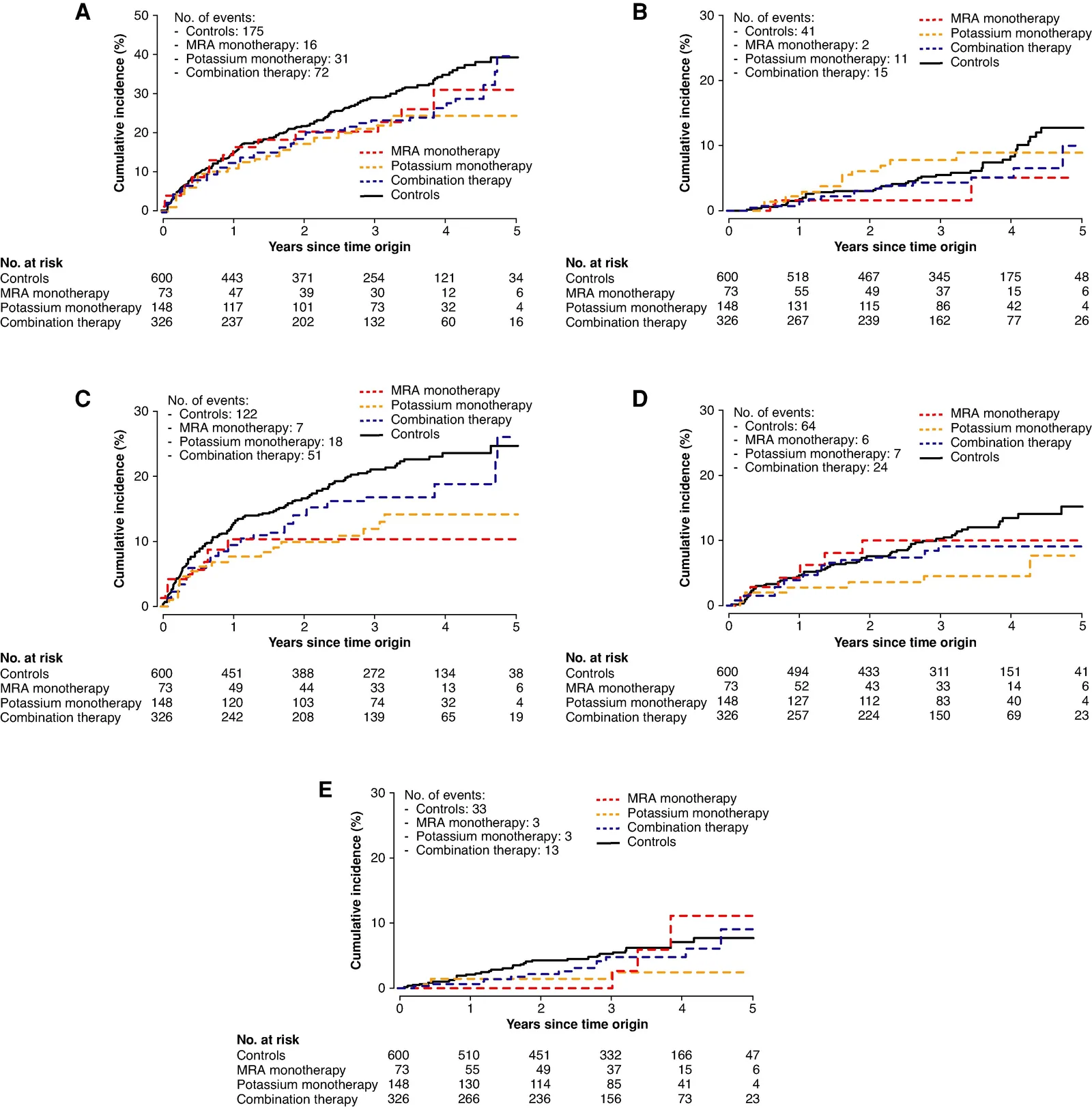
Cumulative incidence of the composite primary endpoint and its individual components. Composite primary endpoint (*A*) and its components. Death from any cause (*B*). Appropriate ICD therapy or VT with a rate of more than 125 beats per minute lasting more than 30 s, documented by electrocardiography or the ICD cardiac electrogram (*C*). Hospitalization for cardiac arrhythmia (*D*). Hospitalization for heart failure (*E*). MRA monotherapy (dashed red), potassium supplement monotherapy (dashed orange), combination therapy (dashed blue), and controls (solid black). For the control group, the time origin was the day of randomization, while the time origin of the three plasma potassium-increasing regimens was the day of ended up-titration. Two sensitivity analyses were conducted to account for this potential immortal time bias and yielded similar findings (see Supplementary material online, *Tables S6* and *S7*). MRA denotes mineralocorticoid receptor antagonist.

*Table 5* provides the unadjusted and adjusted HRs for the endpoints in the control group and the three groups with different plasma potassium-increasing regimens, which shows the trend towards lower HRs for the primary outcomes in each of the three regimens compared to the control group. Findings in the adjusted models were consistent with the unadjusted models, and we detected no significant differences across regimens in both unadjusted and adjusted models. No statistically significant differences in the primary endpoint or any of its components were detected between patients receiving plasma potassium-increasing treatment and those receiving no treatment after a completed up-titration phase. A landmark analysis with 85 days as a landmark time in the control group and an as-treated analysis with treatment exposure as a time-varying variable supported the finding of a numerically lower risk of the endpoints in the three intervention groups compared with the control group and supported the finding of no detectable differences across the three plasma potassium-increasing regimens (see Supplementary material online, *Table S5 and 6*). However, a significant difference in all-cause mortality between the potassium supplement monotherapy group and combination therapy was observed (see Supplementary material online, *Table S6*). Four participants crossed over from combination therapy to potassium supplement monotherapy during follow-up. In three of these participants, MRA was discontinued due to renal insufficiency and malaise as their disease progressed, leading to their death (one due to worsening heart failure and two due to cancer). None of the deaths in the potassium supplement monotherapy group was caused by hyperkalemia. These findings suggest that the significant difference between the combination therapy and potassium monotherapy regimens reflects discontinuation of MRA due to progression of the underlying disease rather than a harmful effect of potassium supplements *per se*.

**Table 5:** The primary composite endpoint and its components

	No. of events	Unadjusted hazard ratio (95% CI) ^a^	Adjusted hazard ratio (95% CI) ^a^
**Primary Composite Endpoint**			
Controls	**175**	Ref.	Ref.
MRA monotherapy	**16**	0.81 (0.49–1.35)	0.80 (0.48–1.35)
Potassium supplement monotherapy	**31**	0.69 (0.47–1.01)	0.66 (0.44–0.98)
Combination therapy	**72**	0.78 (0.59–1.03)	0.69 (0.53–0.92)
**All-cause mortality**			
Controls	**41**	Ref.	Ref.
MRA monotherapy	**2**	0.47 (0.11–1.95)	0.56 (0.13–2.39)
Potassium supplement monotherapy	**11**	1.13 (0.58–2.20)	1.01 (0.49–2.10)
Combination therapy	**15**	0.76 (0.42–1.37)	0.67 (0.37–1.22)
**Appropriate ICD therapy**			
Controls	**122**	Ref.	Ref.
MRA monotherapy	**7**	0.49 (0.23–1.06)	0.44 (0.20–0.94)
Potassium supplement monotherapy	**18**	0.59 (0.36–0.96)	0.60 (0.36–1.01)
Combination therapy	**51**	0.79 (0.57–1.10)	0.68 (0.49–0.96)
**Hospitalization for arrhythmia**			
Controls	**64**	Ref.	Ref.
MRA monotherapy	**6**	0.86 (0.37–1.98)	0.86 (0.37–2.03)
Potassium supplement monotherapy	**7**	0.43 (0.20–0.95)	0.36 (0.16–0.81)
Combination therapy	**24**	0.74 (0.46–1.18)	0.73 (0.46–1.18)
**Hospitalization for heart failure**			
Controls	**33**	Ref.	Ref.
MRA monotherapy	**3**	0.83 (0.25–2.69)	0.92 (0.27–3.12)
Potassium supplement monotherapy	**3**	0.37 (0.11–1.19)	0.33 (0.10–1.13)
Combination therapy	**13**	0.80 (0.42–1.51)	0.70 (0.37–1.36)

Unadjusted and adjusted HRs including 95% CIs for the primary composite endpoint and its components. The HRs for the components of the primary composite endpoint (except all-cause mortality) are cause-specific HRs calculated with a Cox proportional hazards model treating all-cause mortality as a competing event. Adjusted models included the following covariates: baseline plasma potassium level, an estimated glomerular filtration rate <60 mL/min/1.73 m^2^ at baseline, baseline use of potassium supplements, a mineralocorticoid receptor antagonist, or a sodium-glucose cotransporter 2 inhibitor, the indication for an ICD, and previous ventricular tachycardia ^a^ There were no significant differences between intervention groups (*P* ≥ 0.05 for differences between treatment groups). CI denotes confidence interval.

During the study period, 41% of participants in MRA monotherapy, 39% of participants in potassium supplement monotherapy, and 31% of participants in combination therapy had at least one cross-over between regimens. The occurrence of cross-overs was not significantly different across treatment groups (*P* > 0.05). The direction of the cross-overs in each plasma potassium-increasing regimen is indicated in Supplementary material online, *Figure S5*, showing that the majority of the total follow-up time is spent in the regimen assigned at the end of up-titration but with 16–25% of the time spent in another treatment regimen. In addition, participants in combination therapy spent less time without active study medication than those assigned to the monotherapy regimens. The sensitivity analysis comparing the study endpoints with treatment as a time-varying variable (see Supplementary material online, *Table S6*) vs. the analysis based on treatment groups defined at end of up-titration (*Table 5*) produced estimates of a similar direction and magnitude to the primary analysis, supporting the robustness of the findings.

### Safety

Safety outcomes are summarized in *Figure 3* and Supplementary material online, *Figure S6*. During the trial, plasma creatinine levels increased modestly in all intervention regimens as well as in the control group. Overall, at 6 months follow-up, plasma creatinine had increased by 3.5 µmol/L (95% CI, 2.4 to 4.6; *P* < 0.001) in the 572 participants in the intervention group compared to 1.5 µmol/L (95% CI, 0.5 to 2.4; *P* = 0.004) in controls. There was no evidence of difference in the increase in plasma creatinine over the entire study period between the three treatment regimens (see Supplementary material online, *Figure S6*). Likewise, no detectable difference was observed in the composite safety endpoint of all-cause mortality, hospitalization for electrolyte disturbances or renal failure between the overall intervention group and the control group (HR 1.02, 95% CI, 0.68 to 1.55) and no statistically detected difference among the three regimens (*P* = 0.4), although the regimen-specific estimates were imprecise. Looking only at the risk of hospitalization for electrolyte disturbances or renal failure, a total of 17 events occurred in 17 participants (3%) in the intervention group, yielding a cause-specific HR of 1.98 (95% CI, 0.90 to 4.32) in the overall intervention group compared to the control group (*Figure 3*). We did not observe any statistically significant differences in the risk of reaching a safety endpoint among the three regimens: MRA monotherapy: HR 3.02 (95% CI, 0.83 to 11.04); potassium supplement monotherapy: HR 2.63 (95% CI, 0.95 to 7.30); and combination therapy: HR 1.61 (95% CI, 0.63 to 4.07). Adjusted and as-treated analyses yielded comparable results with no detected significant differences between intervention regimens. No hospitalizations for electrolyte disturbances or renal failure occurred during the up-titration phase. Details regarding the safety hospitalizations are provided in Supplementary material online, *Table S7*. No treatment-related deaths occurred during the trial.

**Figure 3:**
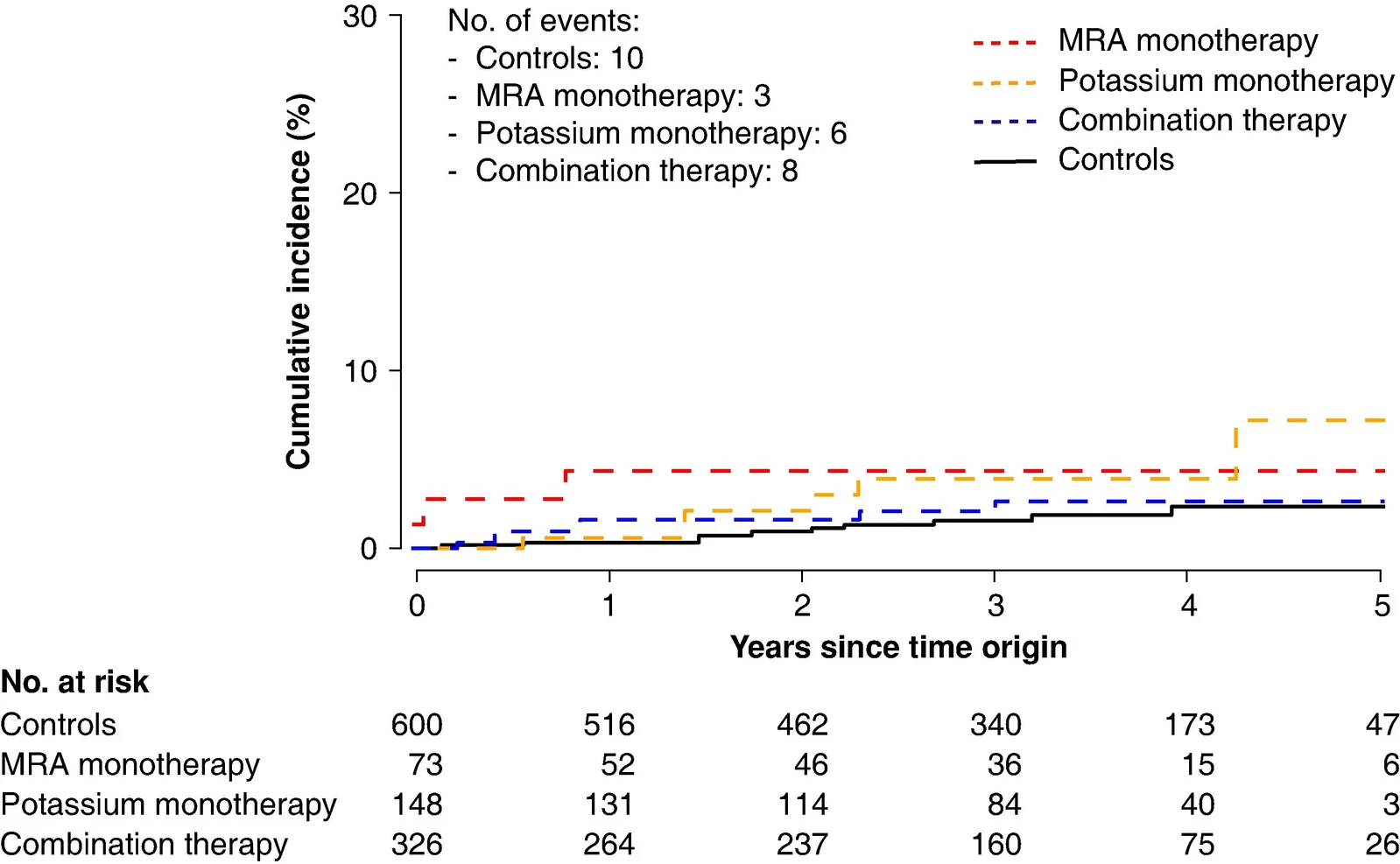
Cumulative incidence of safety outcomes during the study by treatment regimen. Cumulative incidence of hospitalization for hypokalemia, hyperkalemia, or renal failure. MRA monotherapy (dashed red), potassium supplement monotherapy (dashed orange), combination therapy (dashed blue), and controls (solid black). For the control group, the time origin was the day of randomization, while the time origin of the three plasma potassium-increasing regimens was the day of ended up. No safety events occurred in the three plasma potassium-increasing regimens during up-titration, and an as-treated analysis yielded similar results to account for potential immortal time bias. MRA denotes mineralocorticoid receptor antagonist.

### Hypo- and hyperkalemic episodes during follow-up

During the study period, hypokalemic episodes (plasma potassium level <3.5 mmol/L) occurred in 13% of control participants (mean 3.25 mmol/L, range 2.50 to 3.40) and in 6% of the intervention participants (mean 3.24, range 2.60 to 3.40), corresponding to a relative risk of 0.45 (95% CI, 0.30 to 0.66; *P* < 0.001). Hypokalemic episodes occurred in 2 participants (3%) in the MRA monotherapy group (one at 2.70 and one at 3.40 mmol/L), 14 participants (9%) in the potassium supplement monotherapy group (mean 3.27 mmol/L, range 2.60 to 3.40) and 16 participants (5%) in the combination therapy group (mean 3.23 mmol/L, range 2.60 to 3.40) with no evidence of differences in the risk of hypokalemic episodes among the three regimens.

Hyperkalemic episodes (plasma potassium ≥5.5 mmol/L) occurred in one participant in the control group (0.2%) (5.50 mmol/L) and in 18 participants (3%) in the overall intervention group (mean 5.71 mmol/L, range 5.50 to 6.20) (relative risk, 19.7; 95% CI, 2.6 to 147.1; *P* < 0.001). Hyperkalemic episodes occurred in none in the MRA monotherapy group, 5 participants (3%) in the potassium supplement monotherapy group (mean 5.78 mmol/L, range 5.50 to 6.20), and 13 participants (4%) in the combination therapy group (mean 5.68 mmol/L, range 5.50 to 6.0). The risk of hyperkalemia did not seem to differ across the three intervention regimens, but the analyses were underpowered to exclude clinically meaningful differences. None of these episodes resulted in hospitalization for hyperkalemia. The majority of hyperkalemic episodes were managed with an additional subacute confirmatory blood sample, and 12 cases (67%) had a subsequent dose reduction. One patient was admitted with a measured plasma potassium level of 6.2 mmol/L but was discharged shortly after, as the confirmatory test indicated an erroneous initial measurement.

## Discussion

In this predefined subgroup analysis of the POTCAST trial, we sought to compare the efficacy and safety of the plasma potassium-increasing regimens used to target high-normal plasma potassium levels. All three regimens (MRA monotherapy, potassium supplement monotherapy, and combination therapy) significantly increased and maintained stable plasma potassium levels compared with controls. There was no evidence of differences in the primary composite or safety endpoints among the three treatment regimens, although the estimates were imprecise. Compared with controls, intervention participants experienced fewer hypokalemic episodes at the expense of more hyperkalemic episodes.

For MRAs as monotherapy, plasma potassium increased by an average of 0.15 mmol/L at 6 months, in line with prior landmark MRA trials in heart failure, including RALES,^10^ EPHESUS,^11^ and EMPHASIS-HF,^14^ which reported mean increases in serum potassium from 0.1 to 0.3 mmol/L.

Potassium supplements as monotherapy were similarly effective at increasing potassium levels, yielding a mean increase of 0.25 mmol/L at the first 6-month follow-up visit. This finding suggests potassium supplements as a simple, inexpensive, and effective strategy to target higher plasma potassium levels. To our knowledge, this is the first study to evaluate the long-term effects of potassium supplements on plasma potassium levels in patients with cardiovascular diseases, as other trials had short follow-up durations and primarily focused on patients with chronic kidney failure.^15,16^

Combination therapy with MRAs and potassium supplements induced the largest increase in plasma potassium levels with an average increase of 0.36 mmol/L at 6 months, which remained stable throughout follow-up. However, because regimen allocation was not randomized and baseline potassium differed among groups, this finding should not be interpreted as establishing comparative pharmacological potency or a causal additive effect.

Taken together, sustained increases in plasma potassium levels were observed in all three regimens, with mean rises ranging from 0.15 to 0.36 mmol/L at 6 months. Interestingly, although the protocol targeted plasma potassium levels of 4.5–5.0 mmol/L, mean plasma potassium levels remained below this range in all three treatment regimens, and less than half of the participants reached the actual target range at each follow-up visit. Nevertheless, the overall distribution shifted towards higher potassium levels. Given the variability in individual treatment response, more aggressive titration might have increased target attainment but also shifted the upper tail beyond the desired range, increasing the risk of hyperkalaemia. Thus, the observed reduction in clinical events may relate to an upward shift in plasma potassium levels together with prevention of hypokalaemic episodes, rather than sustained achievement of a prespecified protocol target. The reduction in hypokalemia from 13% to 6% was accompanied by an increase in hyperkalemia from 0.2% in the control group to 3% in the intervention group.

In the POTCAST trial, the plasma potassium up-titration algorithm (see Supplementary material online, *Figure S1*) prioritized initiation of MRAs before the introduction of potassium supplements. The present study suggests that potassium supplement monotherapy represents an effective strategy for increasing plasma potassium levels. We detected no statistically significant differences in the primary composite outcome or safety outcomes between treatment regimens. Accordingly, a simplified up-titration algorithm is proposed (*Figure 4*), in which MRAs and potassium supplements are presented as alternative therapeutic options for potassium optimization. MRAs and potassium supplements are shown as potential potassium-raising options, with treatment selection guided by clinical indications, contraindications, tolerability, and participant preference. Potassium supplementation should not replace guideline-directed MRA therapy when an MRA is indicated and tolerated.

**Figure 4:**
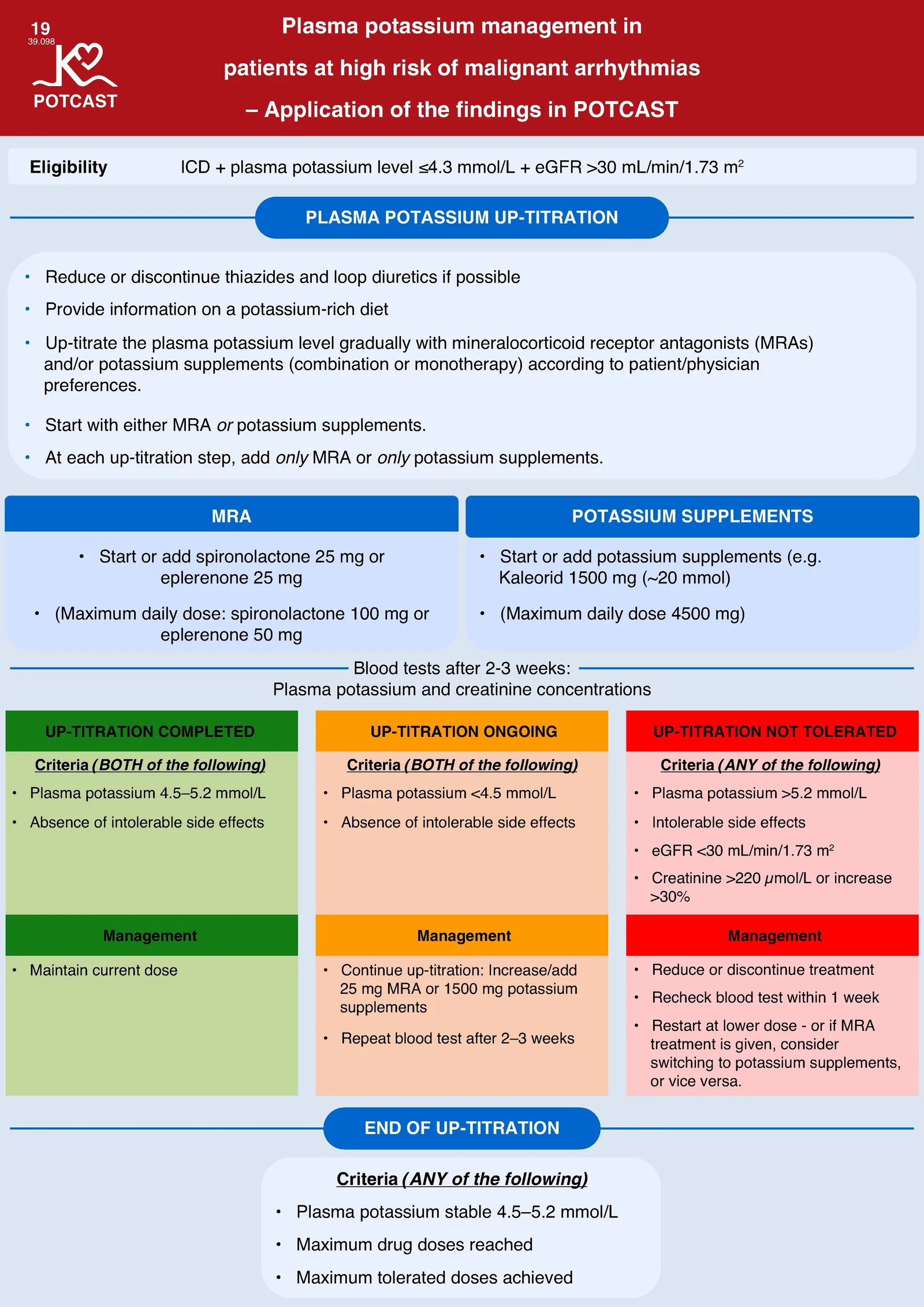
Plasma potassium up-titration algorithm. Proposed simplified plasma potassium up-titration algorithm based on the pharmacological regimens used in the present subgroup analysis. The algorithm presents MRAs and potassium supplements as alternative approaches for plasma potassium up-titration. eGFR denotes estimated glomerular filtration rate. ICD denotes implantable cardioverter-defibrillator. It does not imply comparative efficacy, safety, or therapeutic equivalence between MRA therapy and potassium supplementation but is based on the observed patterns of plasma potassium up-titration in this subanalysis.

In the POTCAST trial, cross-over resulted in participants spending 16–25% of the total follow-up time in regimens other than the one assigned at the end of up-titration. This reflects the real-world setting of the trial, as indications for study medication may change during follow-up because of clinical considerations. Nevertheless, the consistency of the findings between the as-treated and primary analyses indicates that cross-overs have not impacted the results of the study. However, because treatment changes may have occurred in response to clinical deterioration, the analysis remains susceptible to confounding and should be regarded as supportive rather than definitive.

In landmark MRA trials in patients with heart failure,^10,11^ a large proportion of outcome reductions were attributable to fewer sudden cardiac deaths. Since these events are commonly triggered by ventricular arrhythmias, the risk reduction may have been related to an improved potassium homeostasis. Indeed, a large meta-analysis of the trials suggested that part of the observed benefit of MRA treatment in patients with heart failure may stem from increased plasma potassium levels, noting that the MRA-induced decrease in renal potassium loss and the secondary increase in plasma potassium may stabilize the ventricular action potential and reduce arrhythmic vulnerability.^17^ Another interesting observation from these former landmark trials in heart failure supporting the hypothesis of the increase in plasma potassium as a significant contributor to risk reduction of cardiovascular outcomes, such as hospitalization for heart failure or sudden cardiac death, is the relatively early separation of the survival curves, which occurred well before the expected timeframe in which prevention of myocardial fibrosis or other potential structural benefits of MRA therapy would be expected to manifest.^18^ This temporal pattern raises the hypothesis that part of the observed benefit of MRA treatment may be mediated through short-term electrophysiological effects related to improved potassium homeostasis. Evidence from large heart failure trials further supports this concept:

Evidence from contemporary heart failure trials further underscores the clinical importance of preventing hypokalaemia. Post hoc analyses of FINEARTS-HF^19^ showed that finerenone reduced hypokalaemia and that its clinical benefit was maintained even among participants whose serum potassium increased to >5.5 mmol/L. Moreover, in PARAGON-HF,^20^ hypokalemia was associated with a 35% higher risk of worsening heart failure or cardiovascular death compared with potassium levels >5.5 mmol/L. In our study, the frequency of hypokalemic episodes was reduced by more than 50% in the overall intervention group, which may have added to the positive findings. While early separation of event curves may reflect the immediate prevention of hypokalemic episodes, findings from a mechanistic substudy of the POTCAST trial^21^ suggest that the benefits of plasma potassium-targeted therapy may be further amplified over months as total-body potassium gradually increases, reflecting changes in intracellular potassium homeostasis. The EHRA consensus statement provides guidance on hypo- and hyperkalemia during electrical storm but not on low-normal potassium.^22^ Although POTCAST did not investigate electrical storm, its findings raise the possibility that careful optimization towards the high-normal range could complement established management, a hypothesis requiring prospective evaluation.

The duration of the up-titration phase in the POTCAST trial was reported to last a median of 85 days^9^ and included ∼3–4 visits. It should be noted that most ‘visits’ during the up-titration phase consisted of brief telephone contacts addressing blood pressure, potential side effects, and dose adjustments. Prior to these contacts, participants had self-booked blood sampling locally to guide treatment adjustments. According to the study protocol, blood sampling was scheduled every 2 weeks after each increase in study dose, and once targeted levels were attained or participants had reached maximum tolerated doses. This was followed by one confirmatory blood sample after 4 weeks to ensure stable plasma potassium levels and absence of hyperkalaemia.

Overall, our study suggests that a protocolized potassium-targeting strategy can be implemented using MRA therapy only, potassium supplements only, or their combination. The study did not detect any statistically significant differences in clinical outcomes between the treatment regimens in this observational subgroup analysis, and safety outcomes were infrequent. However, the estimates were imprecise, particularly for MRA monotherapy, and should not be interpreted as demonstrating comparative efficacy, safety, or equivalence. The broadly consistent findings observed with potassium supplements and MRA-based regimens suggest that potassium-targeting strategies may be applicable beyond patients with heart failure. These findings are consistent with, but do not independently establish, the hypothesis that low-normal plasma potassium is a modifiable risk factor for ventricular arrhythmic events in patients with an ICD.

### Limitations

Findings from this analysis should be interpreted with certain limitations. First, the choice of treatment regimen within the intervention arm was not randomized, and participant preferences and tolerability determined which plasma potassium-raising regimen was chosen, introducing potential confounding. This was reflected by baseline imbalances between the groups. To account for potential confounding, adjusted Cox proportional hazards models were performed. Adjustment did not materially change the point estimates. However, multivariable adjustment cannot eliminate confounding arising from the non-randomized regimen selection, and residual confounding cannot be excluded. Second, subgroup sizes were modest, particularly in the MRA monotherapy group, limiting statistical power and rendering the results to explorative rather than causal. Third, participants with baseline plasma potassium levels >4.3 mmol/L or advanced kidney impairment (eGFR <30 mL/min/1.73 m^2^) were excluded, and the study included only adults. Therefore, the results are not generalizable to these populations or to pediatric patients with ICDs, in whom arrhythmia burden and ICD therapies differ from those observed in adults.^23^ Finally, plasma potassium levels were closely monitored at repeated study visits within the framework of a protocolized up-titration regimen, creating a controlled environment that may represent a best-case scenario and may not fully reflect routine clinical practice where surveillance and dose adjustments are less stringent.

## Conclusion

In participants at high risk of ventricular arrhythmias treated with an ICD, three pharmacological regimens were associated with sustained increases in plasma potassium levels. All three regimens were associated with favourable clinical outcomes compared to the control group; clinically serious safety events were infrequent, and no clear differential efficacy or safety signal was detected. These observations suggest that plasma potassium-targeting treatment can be achieved through multiple approaches.

## Supplementary Material

euag272_Supplementary_Data

## Data Availability

The data underlying this article contain sensitive patient information and are not publicly available. Anonymized data may be obtained from the corresponding author upon reasonable request and with appropriate approvals.
