# Supplementary material for: Potassium-increasing regimens in patients at high risk of ventricular arrhythmias: a prespecified observational analysis of the randomized POTCAST trial
Source: Europace. 2026 Oct 6;28(10):euag272. doi: 10.1093/europace/euag272 (PMC13688679; doi:10.1093/europace/euag272)
Supplement: Supplementary file 1 [file euag272_supplementary_data.docx]

**Supplementary material**

[Supplementary Figures	2](#_Toc239327576)

[Supplementary Figure 1 Algorithm describing the up-titration process for mineralocorticoid receptor antagonists and potassium supplements, including criteria for dose adjustments, timing of blood sampling, and conditions for completing up-titration	2](#_Toc239327577)

[Supplementary Figure 2 Enrolment, randomization, and follow-up of the participants in the POTCAST trial	3](#_Toc239327578)

[Supplementary Figure 3 Overview of Baseline to End of Up-Titration Medicine Status	4](#_Toc239327579)

[Supplementary Figure 4 Proportion of Participants Achieving Target Potassium Range	5](#_Toc239327580)

[Supplementary Figure 5 Time spent in each regimen by treatment group at end of up-titration	6](#_Toc239327581)

[Supplementary Figure 6 Creatinine Levels During the Study by Treatment Regimen	7](#_Toc239327582)

[Supplementary Tables	8](#_Toc239327583)

[Supplementary Table 1 Reasons to end up-titration receiving only MRA	8](#_Toc239327584)

[Supplementary Table 2 Reasons to end up-titration receiving only potassium supplements despite MRAs being first line therapy in the protocol	8](#_Toc239327585)

[Supplementary Table 3 Reasons for down titration or discontinuation of study medication during the up-titration phase	9](#_Toc239327586)

[Supplementary Table 4 Plasma potassium levels (mmol/L) among the three potassium increasing regimens during the study period	10](#_Toc239327587)

[Supplemental Table 5 Sensitivity analysis using day 85 as time zero for control participants and end of up-titration as time zero for the three plasma potassium increasing regimens	11](#_Toc239327588)

[Supplementary Table 6 Sensitivity analysis with treatment group as a time-varying variable	12](#_Toc239327589)

[Supplementary Table 7 Safety hospitalizations in the control group and three potassium increasing regimens	13](#_Toc239327590)

# Supplementary Figures

## **Supplementary Figure 1** Algorithm describing the up-titration process for mineralocorticoid receptor antagonists and potassium supplements, including criteria for dose adjustments, timing of blood sampling, and conditions for completing up-titration

Legend for supplementary Figure 1: eGFR denotes estimated glomerular filtration rate, o.d. denotes once daily, G.P denotes general practitioner, tid denotes twice daily

## **Supplementary Figure 2** Enrolment, randomization, and follow-up of the participants in the POTCAST trial

Legend for supplementary Figure 2: Flow diagram showing the enrolment, randomization and follow-up of the participants in the POTCAST trial. MRA denotes mineralocorticoid receptor antagonist.

## **Supplementary Figure 3** Overview of Baseline to End of Up-Titration Medicine Status

Legend for supplementary Figure 3: Alluvial diagram illustrating changes in potassium-increasing therapy among the patients in the intervention arm of the POTCAST trial from baseline to the end of the up-titration phase. Each flow represents the number of patients transitioning between treatment categories: MRAs, potassium supplements, combination therapy or no treatment. At baseline, most patients received no potassium-increasing treatment (n=272), whereas at the end of up-titration, the majority received combination therapy (n=326). MRA denotes mineralocorticoid receptor antagonist.

## **Supplementary Figure 4** Proportion of Participants Achieving Target Potassium Range

Legend for supplementary Figure 4: Proportion of participants in each treatment regimen reaching and maintaining potassium levels within the predefined target range during the study. MRA monotherapy (dashed red), potassium supplement monotherapy (dashed orange), combination therapy (dashed blue), and controls (solid black). * p < 0.05 between treatment groups. Significant differences were observed between potassium supplement monotherapy and combination therapy at 6 and 12 months, and between MRA monotherapy and the other treatment regimens at 36 months. MRA denotes mineralocorticoid receptor antagonist.

## **Supplementary Figure 5** Time spent in each regimen by treatment group at end of up-titration

Legend for Supplementary Figure 5: Time spent in each potassium increasing regimen or with no active study medication calculated as the percentage of time in each treatment regimen out of the total observation time in each treatment group. MRA denotes mineralocorticoid receptor antagonist

## **Supplementary Figure 6** Creatinine Levels During the Study by Treatment Regimen

Legend for Supplementary Figure 6: Mean plasma creatinine levels (±95% confidence intervals) during the study. MRA monotherapy (dashed red), potassium supplement monotherapy (dashed orange), combination therapy (dashed blue), and controls (solid black). MRA denotes mineralocorticoid receptor antagonist.

# Supplementary Tables

## **Supplementary Table 1** Reasons to end up-titration receiving only MRA

| **Reason** | **No. of participants (%)** |
| --- | --- |
| Participants reached target potassium levels on MRAs alone | 33 (45) |
| Adverse effects to potassium supplements during up-titration | 25 (34) |
| Did not wish to undergo further up-titration, either because of medication burden or the need for additional blood sampling | 15 (21) |
| Total | 73 (100) |

Legend for supplementary table 1: MRA denotes mineralocorticoid receptor antagonist.

## **Supplementary Table 2** Reasons to end up-titration receiving only potassium supplements despite MRAs being first line therapy in the protocol

| **Reason** | **No. of participants (%)** |
| --- | --- |
| Preference for potassium supplements | 54 (36) |
| Adverse effects/contraindications to MRA during up-titration | 58 (39) |
| Contraindication to MRA at baseline | 30 (20) |
| Other reasons | 6 (4) |
| Total | 148 (100) |

 Legend for supplementary table 2: MRA denotes mineralocorticoid receptor antagonist.

## **Supplementary Table 3** Reasons for down titration or discontinuation of study medication during the up-titration phase

| **Reason** | **Relation to study medication** | |
| --- | --- | --- |
|  | **Mineralocorticoid receptor antagonists**  (N=156) | **Potassium supplements**  (N=99) |
| Due to side effects |  |  |
| Dizziness or hypotension | 47 (30) | - |
| Renal function impairment | 31 (20) | - |
| Gastrointestinal side effects | 12 (8) | 35 (35) |
| Muscle cramps | 10 (6) | - |
| Increased diuresis | 9 (6) | 1 (1) |
| Fatigue | 8 (5) | 3 (3) |
| Hyperkalemia (plasma potassium ≥5.5 mmol/L) | 6 (4) | 9 (9) |
| Hyponatremia | 3 (2) | - |
| Hyperglycemia | 1 (1) | - |
| Hyperlipidemia | 1 (1) | - |
| Gynecomastia | 3 (2) | - |
| Sexual dysfunction | 3 (2) | - |
| Dyspnea | 1 (1) | - |
| Headache | 1 (1) | 2 (2) |
| Mood swings | 1 (1) | - |
| Syncope | 1 (1) | - |
| Myalgia | - | 2 (2) |
| Skin reactions | - | 2 (2) |
| Taste disturbances | - | 1 (1) |
| Pain | - | 1 (1) |
| Malaise | - | 1 (1) |
| At treating physician’s discretion due to rapidly rising plasma potassium levels | 1 (1) | 14 (14) |
| Due to inconvenience related to the medication load | 6 (4) | 13 (13) |
| Due to patient concerns about potential side effects | 3 (2) | 1 (1) |
| Due to non-medical patient related issues (e.g. pregnancy and other personal issues) | 6 (4) | 9 (9) |
| Due to logistical reasons (e.g. inability to complete required blood tests) | 2 (1) | 1 (1) |
| Reason not reported by the treating physician | - | 4 (4) |

## **Supplementary Table 4** Plasma potassium levels (mmol/L) among the three potassium increasing regimens during the study period

| Biannual follow-up | MRA monotherapy (n=73) | Potassium supplement monotherapy  (n=148) | Combination therapy  (n=326) |
| --- | --- | --- | --- |
| Baseline | 4.12 (4.08–4.16)^a^ | 3.99 (3.95–4.03) | 3.98 (3.95–4.00) |
| 6 months | 4.27 (4.18–4.35) | 4.24 (4.18–4.30)^b^ | 4.34 (4.29–4.38) |
| 12 months | 4.27 (4.18–4.36) | 4.14 (4.08–4.21)^b,c^ | 4.35 (4.31–4.39) |
| 18 months | 4.27 (4.16–4.38) | 4.21 (4.15–4.28)^b^ | 4.30 (4.25–4.35) |
| 24 months | 4.36 (4.27–4.46) | 4.25 (4.18–4.33) | 4.32 (4.28–4.37) |
| 30 months | 4.29 (4.14–4.43) | 4.23 (4.15–4.30) | 4.29 (4.24–4.34) |
| 36 months | 4.36 (4.24–4.48) | 4.25 (4.17–4.33) | 4.27 (4.22–4.32) |
| 42 months | 4.33 (4.16–4.50) | 4.26 (4.15–4.36) | 4.27 (4.20–4.34) |
| 48 months | 4.17 (4.01–4.34) | 4.21 (4.09–4.33) | 4.31 (4.22–4.40) |
| 54 months | 4.40 (4.21–4.59) | 4.34 (4.18–4.51) | 4.26 (4.16–4.36) |
| 60 months | 4.22 (4.01–4.43) | 4.15 (3.83–4.47) | 4.34 (4.23–4.46) |

Legend for supplementary table 4: Values are mean plasma potassium in mmol/L (95 % confidence intervals) at baseline and at the biannual follow-up visits.

^a^ p < 0.05 for MRA monotherapy vs. potassium supplement monotherapy and combination therapy.

^b^ p < 0.05 for potassium supplement monotherapy vs. combination therapy.

^c^ p < 0.05 for potassium supplement monotherapy vs. MRA monotherapy.

MRA denotes mineralocorticoid receptor antagonist.

## **Supplemental Table 5** Sensitivity analysis using day 85 as time zero for control participants and end of up-titration as time zero for the three plasma potassium increasing regimens

|  | No. of events | Unadjusted hazard ratio (95% CI) † | Adjusted hazard ratio (95% CI) † |
| --- | --- | --- | --- |
| Primary Composite Endpoint |  |  |  |
| Controls | **161** | Ref. | Ref. |
| MRA monotherapy | **16** | 0.84 (0.50-1.41) | 0.82 (0.49-1.38) |
| Potassium supplement monotherapy | **31** | 0.71 (0.49-1.05) | 0.72 (0.48-1.07) |
| Combination therapy | **72** | 0.81 (0.61-1.07) | 0.71 (0.54-0.95) |
| All-cause mortality |  |  |  |
| Controls | **41** | Ref. | Ref. |
| MRA monotherapy | **2** | 0.43 (0.10-1.78) | 0.51 (0.12-2.16) |
| Potassium supplement monotherapy | **11** | 1.03 (0.53-2.01) | 0.93 (0.45-1.93) |
| Combination therapy | **15** | 0.69 (0.38-1.24) | 0.61 (0.33-1.11) |
| Appropriate ICD- therapy |  |  |  |
| Controls | **98** | Ref. | Ref. |
| MRA monotherapy | **7** | 0.60 (0.28-1.29) | 0.50 (0.23-1.08) |
| Potassium supplement monotherapy | **18** | 0.71 (0.43-1.17) | 0.83 (0.49-1.40) |
| Combination therapy | **51** | 0.96 (0.68-1.35) | 0.80 (0.57-1.13) |
| Hospitalization for arrhythmia |  |  |  |
| Controls | **61** | Ref. | Ref. |
| MRA monotherapy | **6** | 0.85 (0.37-1.97) | 0.85 (0.36-2.00) |
| Potassium supplement monotherapy | **7** | 0.43 (0.20-0.94) | 0.37 (0.17-0.83) |
| Combination therapy | **24** | 0.73 (0.45-1.17) | 0.71 (0.44-1.15) |
| Hospitalization for heart failure |  |  |  |
| Controls | **31** | Ref. | Ref. |
| MRA monotherapy | **3** | 0.81 (0.25-2.66) | 0.87 (0.26-2.96) |
| Potassium supplement monotherapy | **3** | 0.36 (0.11-1.18) | 0.32 (0.09-1.10) |
| Combination therapy | **13** | 0.78 (0.41-1.49) | 0.70 (0.36-1.35) |

Legend for Supplementary Table 6: Unadjusted and adjusted hazard ratios including 95% confidence intervals for the primary composite endpoint and its components. The hazard ratios for the components of the primary composite endpoint (except all-cause mortality) are cause-specific hazard ratios calculated with a Cox proportional hazards model treating all-cause mortality as a competing event.

In this sensitivity analysis participants in the intervention group entered the analysis after completed up-titration (median 85 days) while control participants entered the analysis 85 days after randomization. Compared with follow-up from randomization, 14 control and 7 intervention participants had experienced a prior primary composite event but were event-free at time zero in the present analysis.

Adjusted models included the following covariates: baseline plasma potassium level, an estimated glomerular filtration rate <60 mL/min/1.73 m^2^ at baseline, baseline use of potassium supplements, a mineralocorticoid receptor antagonist, or a sodium-glucose cotransporter 2 inhibitor, the indication for an Implantable Cardioverter-Defibrillator, and previous ventricular tachycardia

**†** There were no significant differences between intervention groups (p ≥0.05 for differences between treatment groups). CI denotes confidence interval.

## **Supplementary Table 6** Sensitivity analysis with treatment group as a time-varying variable

|  | No. of events | Unadjusted hazard ratio (95% CI) | Adjusted hazard ratio (95% CI) |
| --- | --- | --- | --- |
| Primary Composite Endpoint |  |  |  |
| Controls | **175** | Ref. | Ref. |
| MRA monotherapy | **24** | 0.89 (0.58-1.37) | 0.85 (0.55-1.31) |
| Potassium supplement monotherapy | **33** | 0.76 (0.53-1.11) | 0.74 (0.50-1.09) |
| Combination therapy | **62** | 0.70 (0.52-0.93) | 0.60 (0.45-0.81) |
| All-cause mortality |  |  |  |
| Controls | **41** | Ref. | Ref. |
| MRA monotherapy | **5** | 0.95 (0.38-2.40) | 0.92 (0.36-2.39) |
| Potassium supplement monotherapy | **13** | 1.33 (0.71-2.48)* | 1.16 (0.59-2.27)* |
| Combination therapy | **9** | 0.45 (0.22-0.92)* | 0.40 (0.19-0.83)* |
| Appropriate ICD- therapy |  |  |  |
| Controls | **122** | Ref. | Ref. |
| MRA monotherapy | **14** | 0.71 (0.41-1.24) | 0.65 (0.37-1.14) |
| Potassium supplement monotherapy | **20** | 0.67 (0.42-1.08) | 0.69 (0.42-1.12) |
| Combination therapy | **50** | 0.82 (0.59-1.14) | 0.69 (0.50-0.97) |
| Hospitalization for arrhythmia |  |  |  |
| Controls | **64** | Ref. | Ref. |
| MRA monotherapy | **4** | 0.44 (0.16-1.21) | 0.42 (0.15-1.17) |
| Potassium supplement monotherapy | **9** | 0.58 (0.29-1.17) | 0.52 (0.25-1.06) |
| Combination therapy | **24** | 0.75 (0.47-1.20) | 0.72 (0.45-1.16) |
| Hospitalization for heart failure |  |  |  |
| Controls | **33** | Ref. | Ref. |
| MRA monotherapy | **5** | 1.11 (0.43-2.86) | 1.15 (0.44-3.03) |
| Potassium supplement monotherapy | **5** | 0.62 (0.24-1.59) | 0.59 (0.22-1.58) |
| Combination therapy | **8** | 0.49 (0.23-1.07) | 0.41 (0.19-0.90) |

Legend for Supplementary Table 6: Unadjusted and adjusted hazard ratios including 95% confidence intervals for the primary composite endpoint and its components. The hazard ratios for the components of the primary composite endpoint (except all-cause mortality) are cause-specific hazard ratios calculated with a Cox proportional hazards model treating all-cause mortality as a competing event.

In this sensitivity analysis all participants in both the intervention and control group entered the analysis at the day of randomization. Treatment exposure was modelled as a time-varying covariate.

Adjusted models included the following covariates: baseline plasma potassium level, an estimated glomerular filtration rate <60 mL/min/1.73 m^2^ at baseline, baseline use of potassium supplements, a mineralocorticoid receptor antagonist, or a sodium-glucose cotransporter 2 inhibitor, the indication for an Implantable Cardioverter-Defibrillator, and previous ventricular tachycardia

*A significant difference between the risk of all-cause mortality was observed between the potassium monotherapy regimen and the combination therapy regimen. There were no other significant differences between intervention groups. CI denotes confidence interval.

## **Supplementary Table 7** Safety hospitalizations in the control group and three potassium increasing regimens

|  | **Reason for hospitalization** | **Plasma creatinine level (µmol/L) at admission. (Habitual level)** | **Plasma potassium level (mmol/L) at admission** | **Study drug status before admission (mg)** |
| --- | --- | --- | --- | --- |
| **Controls** | |  |  |  |
| **1** | Hypokalemia | - | 3.1 | ^-^ |
| **2** | Hypokalemia | - | 2.7 | ^-^ |
| **3** | Hypokalemia | - | 3.1 | ^-^ |
| **4** | Hypokalemia | - | 2.5 | ^-^ |
| **5** | Hyperkalemia | - | 5.6 | ^-^ |
| **6** | Hyperkalemia | - | 6.0 | ^-^ |
| **7** | Hypomagnesemia | - | 3.2 | ^-^ |
| **8** | Renal failure | 150 (123) | 4.1 | ^-^ |
| **9** | Renal failure | 380 (137) | 3.2 | ^-^ |
| **10** | Renal failure | 290 (95) | 4.5 | ^-^ |
| **11** | Renal failure | 370 (197) | 5.4 | ^-^ |
| **12** | Renal failure | 430 (83) | 4.3 | ^-^ |
| **MRA monotherapy** | |  |  |  |
| **1** | Hyperkalemia | - | 6.4 | MRA: 50.0 |
| **2** | Hyperkalemia | - | 6.9 | MRA: 25.0 |
| **3** | Renal failure | 770 (130) | 4.7 | MRA: 50.0 |
| **Potassium supplements monotherapy** | |  | | |
| **1** | Hypokalemia | - | 2.7 | Potassium supplements: 4,500 |
| **2** | Hyperkalemia | - | 6.4 | Potassium supplements: 3,000 |
| **3** | Hypocalcemia | - | 3.5 | Potassium supplements: 1,500 |
| **4** | Renal failure | 215 (113) | 4.3 | Potassium supplements: 3,000  MRA: 25.0 |
| **5** | Renal failure | 230 (197) | 4.1 | Potassium supplements: 6,000 |
| **6** | Renal failure | 1600 (106) | 5.3 | Potassium supplementation: 0.0 |
| **Combination therapy** | |  |  |  |
| **1** | Hyperkalemia | - | 6.6 | MRA: 50.0  Potassium supplements: 2,250 |
| **2** | Hyperkalemia | - | 4.9 | MRA: 100.0  Potassium supplements: 4,500 |
| **3** | Hyperkalemia | - | 5.5 | MRA: 50.0  Potassium supplements: 4,500 |
| **4** | Hyperkalemia | - | 6.4 | MRA: 37.5  Potassium supplements: 4,500 |
| **5** | Renal failure | 150 (113) | 4.3 | MRA: 25.0  Potassium supplements: 2,250 |
| **6** | Renal failure | 190 (110) | 5.1 | MRA: 25.0  Potassium supplements: 750 |
| **7** | Renal failure | 324 (230) | 6.0 | MRA: 0.0  Potassium supplements: 1,500 |
| **8** | Renal failure | 340 (74) | 5.0 | MRA: 25.0  Potassium supplements: 1,500 |

Legend for supplementary table 5. Presented according to the intention-to-treat analysis. MRA denotes mineralocorticoid receptor antagonist. Habitual creatinine level defined as the mean value of three preceding outpatient measurements.
